# Rutin-Enriched Extract from *Coriandrum sativum* L. Ameliorates Ionizing Radiation-Induced Hematopoietic Injury

**DOI:** 10.3390/ijms18050942

**Published:** 2017-04-29

**Authors:** Xiaodan Han, Xiaolei Xue, Yu Zhao, Yuan Li, Weili Liu, Junling Zhang, Saijun Fan

**Affiliations:** Tianjin Key Laboratory of Radiation Medicine and Molecular Nuclear Medicine, Institute of Radiation Medicine, Peking Union Medical College and Chinese Academy of Medical Science, Tianjin 300192, China; hanxiaodan1202@gmail.com (X.H.); xuexiaolei1234@gmail.com (X.X.); zhaoyupumc@gmail.com (Y.Z.); hanmeimei1202@gmail.com (Y.L.); liuweili@irm-cams.ac.cn (W.L.)

**Keywords:** ionizing radiation, coriander extract, total body irradiation, hematopoietic stem and progenitor cells, reactive oxygen species

## Abstract

Hematopoietic injury is a major cause of mortality in radiation accidents and a primary side effect in patients undergoing radiotherapy. Ionizing radiation (IR)-induced myelosuppression is largely attributed to the injury of hematopoietic stem and progenitor cells (HSPCs). Coriander is a culinary herb with multiple pharmacological effects and has been widely used in traditional medicine. In this study, flavonoids were identified as the main component of coriander extract with rutin being the leading compound (rutin-enriched coriander extract; RE-CE). We evaluated the radioprotective effect of RE-CE against IR-induced HSPCs injury. Results showed that RE-CE treatment markedly improved survival, ameliorated organ injuries and myelosuppression, elevated HSPCs frequency, and promoted differentiation and proliferation of HSPCs in irradiated mice. The protective role of RE-CE in hematopoietic injury is probably attributed to its anti-apoptotic and anti-DNA damage effect in irradiated HSPCs. Moreover, these changes were associated with reduced reactive oxygen species (ROS) and enhanced antioxidant enzymatic activities in irradiated HSPCs. Collectively, these findings demonstrate that RE-CE is able to ameliorate IR-induced hematopoietic injury partly by reducing IR-induced oxidative stress.

## 1. Introduction

Exposure to ionizing radiation (IR) may induce injury in various tissues and organs, among which bone marrow (BM) is the most radiosensitive tissue [1]. Acute myelosuppression is the primary cause of death after accidental or intentional exposure to a high dose of total body irradiation (TBI) [2,3]. Several studies have demonstrated that IR-induced myelosuppression is mainly due to impaired proliferation and differentiation ability and increased apoptosis and senescence of hematopoietic stem and progenitor cells (HSPCs) [2,3,4,5]. Therefore, it should be a primary goal to protect HSPCs in the development of novel medical countermeasures against IR, and there is a critical need to develop effective radioprotective agents that can ameliorate IR-induced HSPCs injury.

It has been well established that reactive oxygen species (ROS) play a critical role in IR-induced hematopoietic injury [6,7]. IR induces the excessive production of ROS including superoxide, hydroxyl radicals, and hydrogen peroxide derived from the radiolysis of water. Oxidative stress from ROS may induce DNA damage, cell apoptosis, and senescence [5,7,8]. Moreover, ROS have been illustrated in several studies to be responsible for the loss of self-renewing ability and premature exhaustion of hematopoietic stem cells [9,10]. Fortunately, ROS can be eliminated by exogenous administration of antioxidants or by enhancing endogenous antioxidant enzyme activities, such as superoxide dismutase (SOD), glutathione peroxidase (GSH-PX), and catalase (CAT). Antioxidants have been extensively studied as ROS scavengers, which may mitigate the oxidative stress induced by IR [11,12,13,14,15,16].

Coriander (*Coriandrum sativum* L.) is an annual herb belonging to the *Apiaceae* family that has been used as a flavoring agent and traditional remedy. Essential oil, avonoids, phenolic acids, and polyphenols are important constituents of the aerial parts of coriander, and essential oil and fatty oil are the major components of coriander seeds [17,18]. Different parts of coriander have been reported for multiple health functions and biological activities, including antioxidant, antimicrobial, anti-diabetic, antidyslipidemic, anticonvulsant, anxiolytic, diuretic, antihypertensive, anti-inflammatory, and antimutagenic activities [17,18,19,20]. Hwang and his colleagues found that coriander possessed the potential to prevent ultraviolet radiation-induced skin photoaging [21]. More importantly, coriander extracts have been used to scavenge ROS as well as up-regulate endogenous cellular antioxidant systems [22,23]. These findings suggest that coriander may act as a radioprotective agent to mitigate IR-induced hematopoietic injury due to its antioxidant activity.

In this study, we assessed the protective effects of the aqueous and ethanol extract mixture from the aerial parts of coriander on IR-induced hematopoietic injury in a well-established TBI mouse model [12]. Our data showed that rutin-enriched coriander extract (RE-CE) ameliorated myelosuppression, elevated HSPCs frequency, and improved the proliferation and differentiation ability of HSPCs, probably by inhibiting apoptosis and DNA damage in irradiated HSPCs. These protective effects of RE-CE may be attributed to scavenging ROS and activating antioxidant enzymes in irradiated HSPCs. All these findings suggest that CE treatment is able to protect the hematopoietic system from IR-induced injury.

## 2. Results

### 2.1. RE-CE Ameliorates IR-Induced Organ Injury

**Figure 1:**
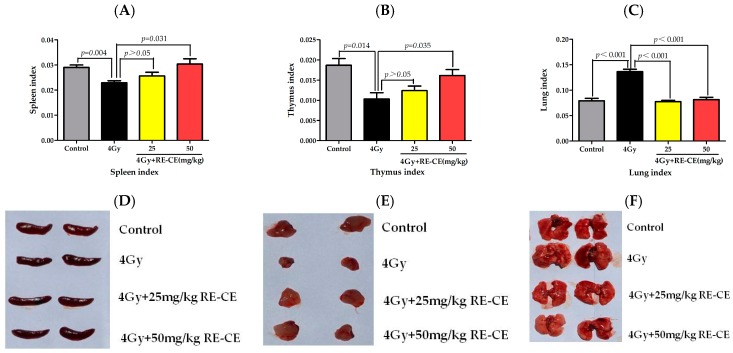
RE-CE rescues organ index in irradiated mice. Mice were daily treated with vehicle or different concentrations of rutin-enriched coriander extract (RE-CE) 30 min before 4 Gy total body irradiation (TBI) and up to 7 days after TBI. Control mice were sham-irradiated. Spleen index (**A**), thymus index (**B**), and lung index (**C**) of mice were calculated on the 14th day after exposure to TBI. 50 mg/kg RE-CE treatment significantly increased the spleen index and thymus index, as well as decreased the lung index of irradiated mice. Representative images of spleen (**D**), thymus (**E**) and lung (**F**) on the 14th day after exposure to TBI are shown. Organ indices are presented as means ± SEM (*n* = 5).

**Figure A1:**
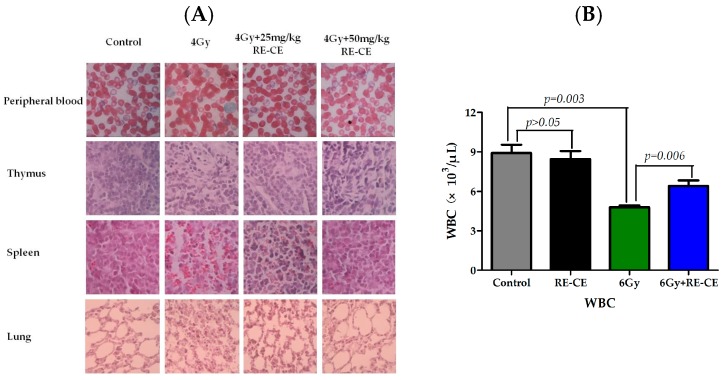

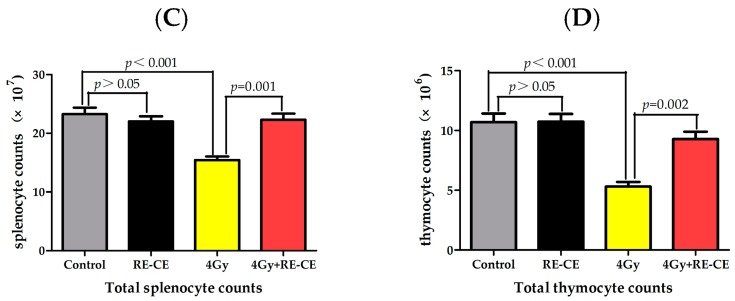
CE treatment ameliorates IR-induced injury in organ and peripheral blood. Mice were daily treated with the vehicle or CE as described in the Materials and Methods. (**A**) Representative H&E stained spleen, thymus, lung, and wright-giemsa stained peripheral blood （×40）on the 14th day after exposure to TBI; (**B**) The number of WBCs in peripheral blood 2 months after 6 Gy TBI; (**C**) Total splenocyte counts on the 14th day after exposure to TBI; (**D**) Total thymocyte counts on the 14th day after exposure to TBI.

It has been found that IR can cause damage to multiple organs, leading to changes of organ indexes, including a decline in the spleen index and thymus index, but a rise in lung index [24,25]. To determine whether RE-CE treatment protected mice from IR-induced organ index changes, mice were exposed to 4 Gy TBI and treated with the vehicle or RE-CE as described in the Materials and Methods. As shown in Figure 1A,B,D,E, 50 mg/kg RE-CE treatment significantly attenuated the reduction in the spleen index and thymus index of irradiated mice, while the 25 mg/kg RE-CE treatment showed a slight effect. Interestingly, the lung index of irradiated mice was markedly decreased by consumption of 50 mg/kg CE as well as 25 mg/kg RE-CE (Figure 1C,F). The thymus and spleen of irradiated mice atrophied and exhibited decreased lymphocytes compared to the controls. Congestion and inflammatory cell infiltration could be observed in the lungs of irradiated mice. CE treatment alleviated these pathological changes (Figure A1). In addition, 50 mg/kg RE-CE treatment attenuated the declines in total splenocyte and thymocyte counts in 4 Gy irradiated mice (Figure A1C,D). These findings suggest that RE-CE plays a protective role in IR-induced organ injury in mice, and the 50 mg/kg RE-CE treatment exhibits higher efficiency than the 25 mg/kg RE-CE treatment.

### 2.2. RE-CE Alleviates IR-Induced Myelosuppression and Promotes Myeloid Skewing Recovery

**Figure 2:**
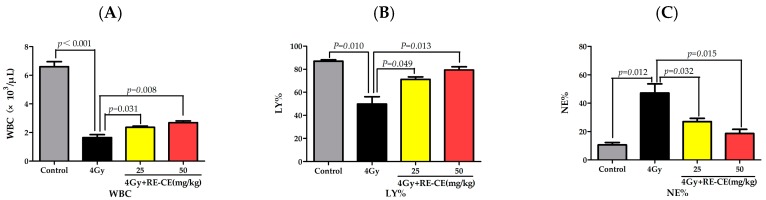

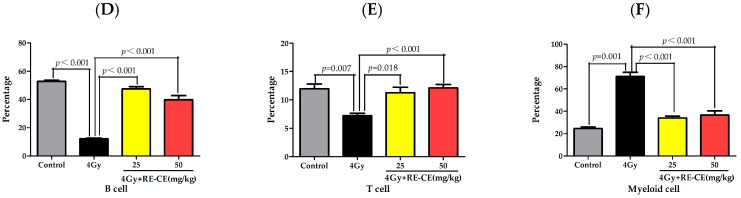
RE-CE alleviates ionizing radiation (IR)-induced myelosuppression and promotes myeloid skewing recovery in irradiated mice. Mice were sham-irradiated as the control or irradiated with 4 Gy TBI after receiving the vehicle or RE-CE treatment as described in the Materials and Methods. The number of white blood cells (WBCs) (**A**), lymphocyte percentage (LY%) (**B**), and neutrophil percentage (NE%) (**C**) in peripheral blood were quantified on the 14th day after exposure to TBI. The percentage of B cells (**D**), T cells (**E**), and myeloid cells (**F**) in peripheral blood were analyzed by Fluorescence-Activated Cell Sorting (FACS) on the 14th day after exposure to TBI. RE-CE treatment significantly elevated WBC counts, LY%, B cell percentage, T cell percentage, and reduced NE% and myeloid cell percentage. All data are presented as means ± SEM (*n* = 5).

It has been well established that IR can induce myelosupression which is characterized by cytopenia accompanied with myeloid skewing in peripheral blood [26,27]. To determine whether RE-CE treatment promoted hematopoietic recovery in irradiated mice, we analyzed the number of different types of peripheral blood cells using a hematology analyzer, and the percentage of B cells, T cells, and myeloid cells using flow cytometry. As illustrated in Figure 2 and Figure A1, irradiated mice exhibited a significant decline in white blood cells (WBCs), lymphocyte percentage (LY%), and percentage of B cells and T cells, but an increase in neutrophil percentage (NE%) and percentage of myeloid cells in peripheral blood at day 14 after 4 Gy TBI, when compared to unirradiated controls. Treatment of 50 mg/kg RE-CE and 25 mg/kg RE-CE elevated WBC counts, LY%, B cell percentage and T cell percentage (Figure 2A,B,D,E and Figure A1), but reduced NE% and myeloid cell percentage (Figure 2C,F and Figure A1) in the peripheral blood of irradiated mice. The decline in WBC count was still found 2 months after 6 Gy TBI, and the 50 mg/kg RE-CE treatment attenuated such a decline (Figure A1B). These results suggest that RE-CE treatment promotes recovery from IR-induced myelosuppression and myeloid skewing to maintain hematopoietic homeostasis.

### 2.3. RE-CE Mitigates IR-Induced Differentiation-Related Dysfunction of B Cells and Erythrocytes in BM

**Figure 3:**
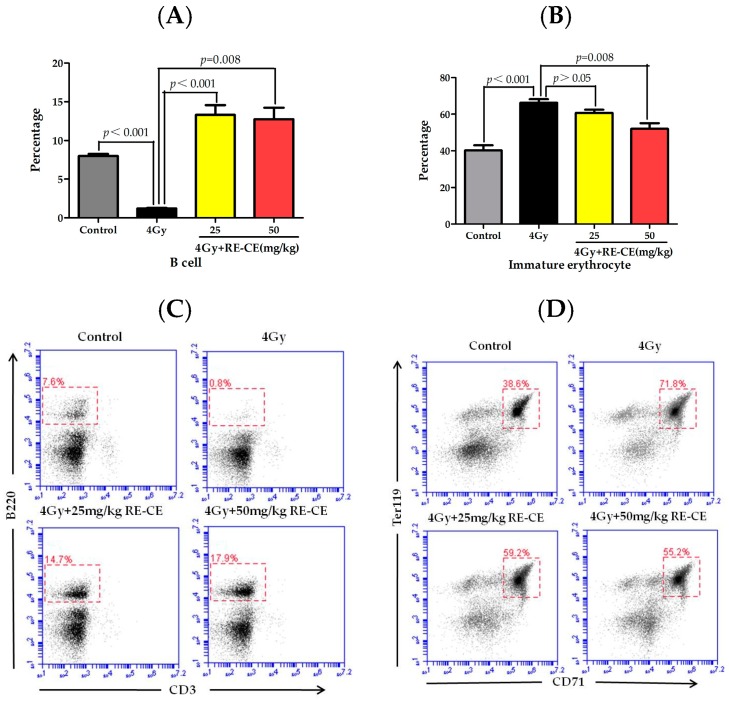
RE-CE mitigates IR-induced differentiation-related dysfunction of B cells and erythrocytes. Mice were sham-irradiated as control or irradiated with 4 Gy TBI after receiving the vehicle or RE-CE treatment as described in the Materials and Methods. B cells percentage (**A**) and CD71^+^Ter119^+^ immature erythrocyte percentage (**B**) in bone marrow (BM) were analyzed by FACS on the 14th day after exposure to TBI. A representative FACS analysis shows the B cells percentage (**C**) and CD71^+^Ter119^+^ immature erythrocyte percentage (**D**) in BM. RE-CE treatment increased the B cell percentage and decreased the CD71^+^Ter119^+^ immature erythrocyte percentage. The data are presented as means ± SEM (*n* = 5).

There was a notable decline in B cell percentage in BM after 4 Gy TBI, but RE-CE treatment increased the B cell percentage (Figure 3A). Additionally, the percentage of CD71^+^Ter119^+^ immature erythrocytes was elevated after 4 Gy TBI, but RE-CE treatment alleviated this effect of IR on immature erythrocytes (Figure 3B).

### 2.4. RE-CE Attenuates IR-Induced Alterations in HSPCs Frequency

**Figure 4:**
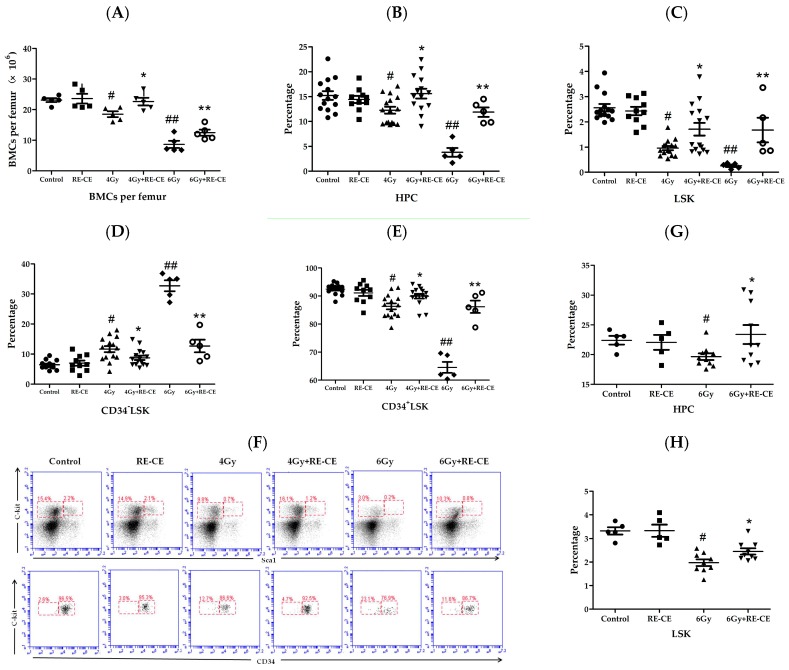

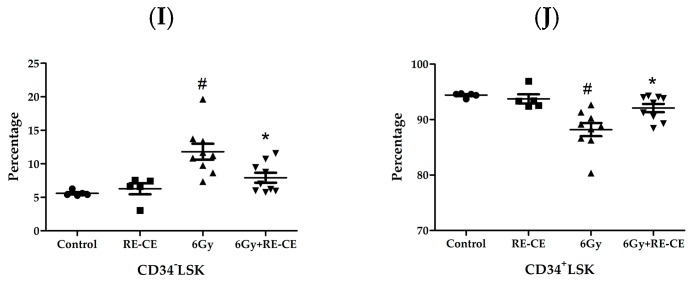
RE-CE attenuates IR-induced alterations in hematopoietic stem and progenitor cells (HSPCs) frequency. Mice were daily treated with the vehicle or 50 mg/kg RE-CE by gavage for 30 min before 4 Gy or 6 Gy TBI and up to 7 days after radiation. Mice were sham-irradiated as the control or CE group. (**A**) The number of BM cells (BMCs) per femur on the 14th day after exposure to 4 Gy or 6 Gy TBI; (**B**–**D**) The percentages of HSPCs were analyzed by FACS on the 14th day after exposure to 4 Gy or 6 Gy TBI; (**B**) The percentage of HPCs (hematopoietic progenitor cells, Lineage^−^sca1^−^c-kit^+^) among the lineage-negative cells; (**C**) The percentage of LSKs (Lineage^−^sca1^+^c-kit^+^) among the lineage-negative cells; (**D**) The percentage of CD34^−^LSKs among the LSK cells; (**E**) The percentage of CD34^+^LSKs among the LSK cells; (**F**) Representative FACS analyses of the percentage of HPCs, LSKs, CD34^+^LSKs, and CD34^−^LSKs; (**G**–**J**) The percentages of HSPCs analyzed by FACS 2 months after exposure to 6 Gy TBI; (**G**) The percentage of HPCs among lineage-negative cells; (**H**) The percentage of LSKs among lineage-negative cells; (**I**) The percentage of CD34^−^LSKs among LSK cells; (**J**) The percentage of CD34^+^LSKs among LSK cells. RE-CE treatment significantly increased the number of BMCs per femur, the frequencies of HPC, LSK, and CD34^+^LSK, and resulted in decreased frequencies of CD34^−^LSK. The data are presented as mean ± SEM (*n* = 5−15); *^#^ p* < 0.05 vs. control; * p < 0.05 vs. 4 Gy.

**Figure A2:**
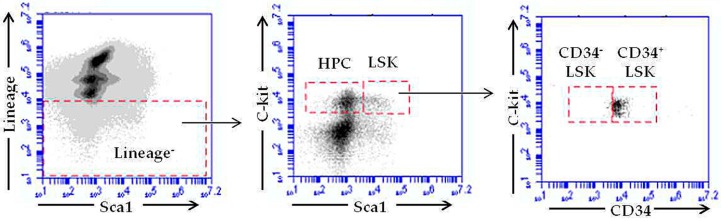
Gating strategy for HSPCs.

Since the exhaustion of HSPCs is a critical cause of myelosuppression, we examined whether RE-CE treatment attenuated the IR-induced decline in BM cells (BMCs) and HSPCs frequency. The 4 Gy and 6 Gy TBI irradiated mice both exhibited decreased BMCs, but RE-CE treatment attenuated these declines in the 4 Gy as well as 6 Gy irradiated mice (Figure 4A). As shown in Figure 4B–F, 4 Gy and 6 Gy TBI both caused a decrease in HPC (hematopoietic progenitor cells, Lineage^−^Scal^−^c-kit^+^), LSK (Lineage^−^Scal^+^c-kit^+^), and CD34^+^LSK (CD34^+^Lineage^−^Scal^+^c-kit^+^) frequency, and an increase in CD34^−^LSK (CD34^−^Lineage^−^Scal^+^c-kit^+^) frequency at day 14 after TBI. Furthermore, HSPCs exhibited a similar trend 2 months after 6 Gy TBI (Figure 4G–J). RE-CE treatment attenuated these effects in HPC (Figure 4B,G), LSK (Figure 4C,H), CD34^+^LSK (Figure 4E,J), and CD34^−^LSK frequency (Figure 4D,I). These results suggest that RE-CE treatment promotes recovery from IR-induced alternations in HSPCs frequency. The complete gating strategy for HSPCs is presented in Figure A2.

### 2.5. RE-CE Improves Colony Forming and Engraftment Abilities of HSPCs in Irradiated Mice

**Figure 5:**
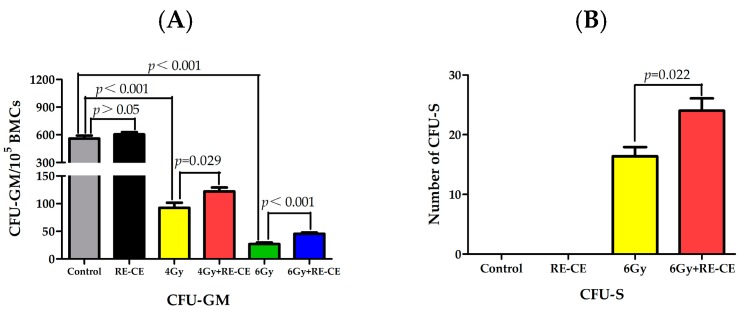

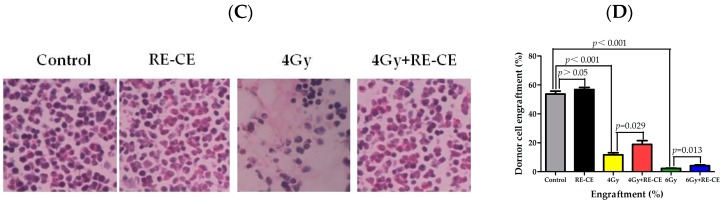
RE-CE improves colony forming and engraftment abilities of HSPCs in irradiated mice. Mice were daily treated with the vehicle or RE-CE as described in the Materials and Methods. (**A**) The number of colonies of granulocyte macrophage cells (CFU-GM) per 10^5^ bone marrow cells (BMCs); (**B**) The number of spleen colony-forming units (CFU-S) on the 14th day after exposure to TBI; (**C**) Representative H&E stained femurs (×40) on the 14th day after exposure to TBI; (**D**) Donor cell engraftment in the peripheral blood of recipients 4 months after competitive bone marrow transplantation. RE-CE treatment significantly increased the number of CFU-GM and CFU-S, and promoted HSPCs colony forming and donor cell engraftment in irradiated mice. The data are presented as means ± SEM (*n* = 5 in panel **A** and **B**, *n* = 10 in panel **C**).

**Figure A3:**
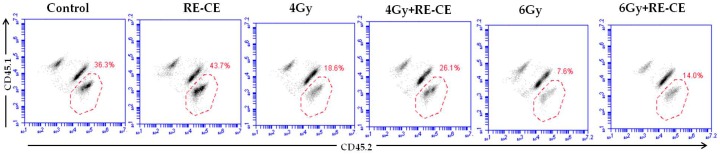
CE treatment promotes donor cell engraftment in irradiated mice. Mice were daily treated with the vehicle or CE as described in the Materials and Methods. A representative analysis of the percentage of donor cell engraftment in the peripheral blood of recipients by flow cytometry.

We conducted colony of granulocyte macrophage cells (CFU-GM) assays, spleen colony-forming units (CFU-S) assays, and competitive bone marrow transplantation to evaluate whether RE-CE treatment improved the colony forming and engraftment abilities of HSPCs in irradiated mice. The results revealed that the CFU-GM number remarkably decreased in irradiated mice compared to control mice, but RE-CE treatment attenuated this decline (Figure 5A). In addition, 6 Gy TBI stimulated the formation of spleen colonies and RE-CE treatment increased the number of CFU-S in irradiated mice (Figure 5B). At day 14 after 4 Gy TBI, hypoplastic bone marrow was observed in irradiated mice, but RE-CE-treated mice exhibited increased cellularity in bone marrow (Figure 5C). More importantly, 4 Gy and 6 Gy TBI both induced a reduction in engraftment after competitive bone marrow transplantation, but RE-CE treatment promoted donor cell engraftment in 4 Gy and 6 Gy irradiated mice 4 months after competitive bone marrow transplantation (Figure 5D and Figure A3). These data demonstrate that RE-CE treatment improves the colony forming and engraftment abilities of irradiated HSPCs.

### 2.6. RE-CE Inhibits IR-Induced Apoptosis and DNA Damage in HSPCs

**Figure 6:**
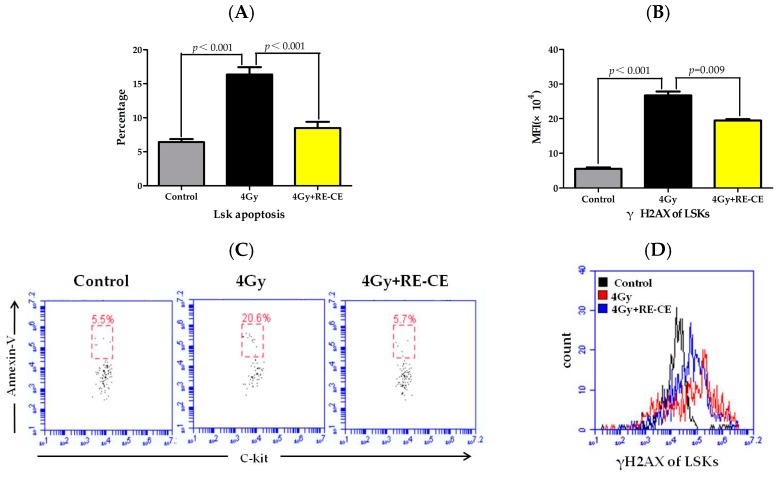
RE-CE inhibits IR-induced apoptosis and DNA damage in LSKs. Mice were daily treated with vehicle or RE-CE as described in the Materials and Methods. (**A**) The percentage of apoptosis in LSKs; (**B**) The mean fluorescence intensity (MFI) of phospho-histone H2AX (γH2AX) in LSKs; (**C**) A representative FACS analysis of the percentage of cell apoptosis in LSKs; (**D**) A representative analysis of γH2AX expression in LSKs by flow cytometry. RE-CE treatment significantly reduced the percentage of cell apoptosis, and decreased the MFI of γH2AX in irradiated LSKs. All data are presented as means ± SEM (*n* = 5).

**Figure A4:**
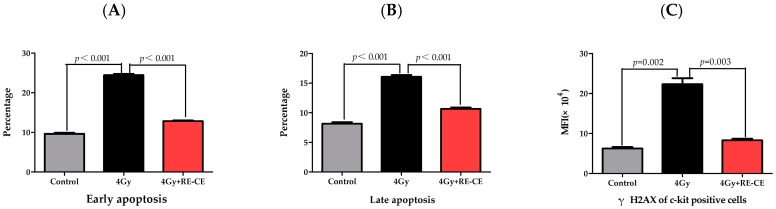

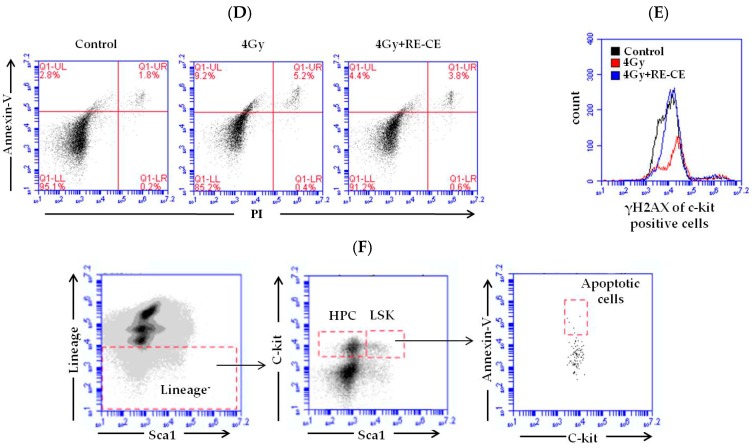
CE treatment inhibits IR-induced apoptosis and DNA damage in HSPCs. Mice were daily treated with the vehicle or CE as described in the Materials and Methods. Early apoptosis (Annexin V^+^PI^−^) (**A**), late apoptosis (Annexin V^+^PI^+^) (**B**) and MFI of γH2AX (**C**) in c-kit positive cells were measured on the 14th day after exposure to TBI; (**D**) A representative FACS analysis of the percentage of apoptosis in c-kit positive cells; (**E**) A representative analysis of γH2AX expression in c-kit positive cells by flow cytometry; (**F**) Gating strategy for the analysis of LSK apoptosis. CE treatment significantly reduced the percentage of cell apoptosis, and decreased the MFI of γH2AX in irradiated c-kit positive cells. All data are presented as means ± SEM (*n* = 5).

IR may induce DNA damage in HSPCs, namely double strand breaks (DSBs), leading to cell apoptosis, dysfunctional cell growth, and ultimately hematopoietic disorders [4]. As shown in Figure 6, there is a noticeable increase in both apoptosis (Annexin V+) and the mean flourescence intensity (MFI) of phospho-histone H2AX (γH2AX) in irradiated LSKs, but RE-CE treatment markedly attenuated these trends (Figure 6). Similar results were obtained in c-kit positive cells (Figure A4). These results suggest that RE-CE promotes hematopoietic recovery probably by inhibiting IR-induced apoptosis and DNA damage in HSPCs.

### 2.7. RE-CE Scavenges IR-Induced ROS in HSPCs

**Figure 7:**
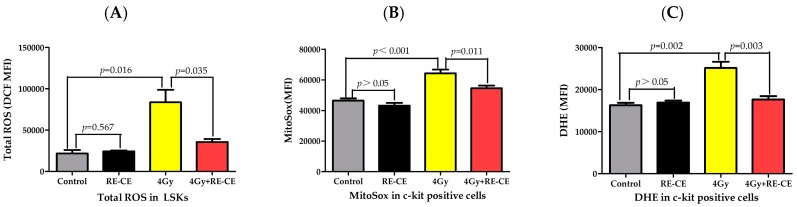
RE-CE scavenges IR-induced reactive oxygen species (ROS) in HSPCs. Mice were daily treated with the vehicle or RE-CE as described in the Materials and Methods. (**A**) The total ROS levels in LSKs were presented as the MFI of 2,7-dichlorodihydrofluorescein diacetate (DCFDA); (**B**) The mitochondria-derived ROS levels in c-kit positive cells were presented as MFI of MitoSox; (**C**) The levels of superoxide free radicals were presented as the MFI of dihydroethidium (DHE). RE-CE treatment significantly decreased the MFI of DCF, MitoSox, and DHE in irradiated HSPCs. The MFI of DCF, MitoSox and DHE are presented as means ± SEM (*n* = 5).

**Figure A5:**
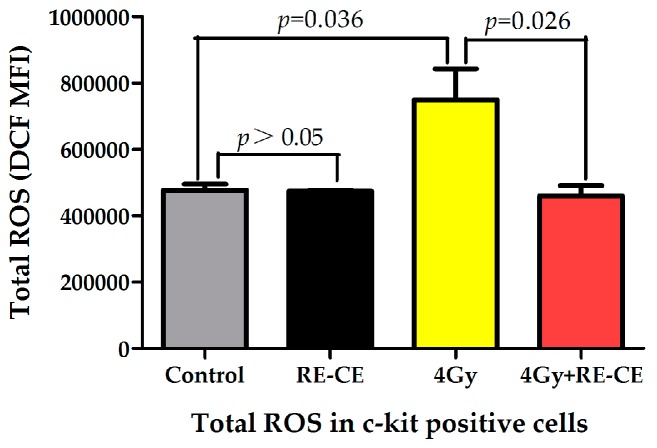
CE scavenges IR-induced ROS in c-kit positive cells. Mice were daily treated with the vehicle or CE as described in the Materials and Methods. The total ROS levels in c-kit positive cells were presented as MFI of DCF. CE treatment significantly decreased the MFI in irradiated c-kit positive cells. The MFI of DCF, are presented as means ± SEM (*n* = 5).

The indirect effect of IR is mainly caused by ROS, which contribute to IR-induced hematopoietic injury. To determine whether RE-CE treatment reduced IR-induced ROS, we measured the MFI of 2,7-dichlorofluorescein (DCF), MitoSox, and dihydroethidium (DHE) in LSKs and c-kit positive cells 14 days after 4 Gy TBI to evaluate the total cellular ROS, mitochondria-derived ROS (superoxide), and superoxide free radicals levels, respectively. The MFI of 2,7-dichlorodihydrofluorescein diacetate (DCFDA) (Figure A5), MitoSox (Figure 7B), and DHE (Figure 7C) in c-kit positive cells was significantly elevated after radiation, but RE-CE treatment attenuated the IR-induced elevation in ROS levels. Similar results of total cellular ROS were observed in LSKs (Figure 7A). These results suggest that RE-CE serves as a free radical scavenger in irradiated mice.

### 2.8. RE-CE Ameliorates IR-Induced Repression of Antioxidant Enzymatic Activities in c-Kit Positive Cells

**Figure 8:**
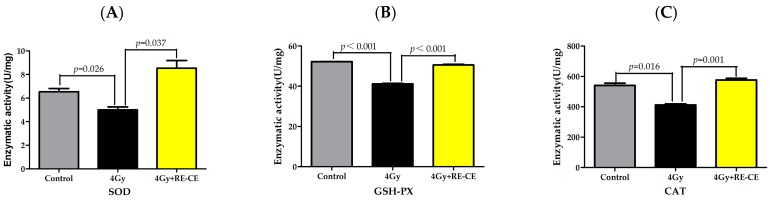

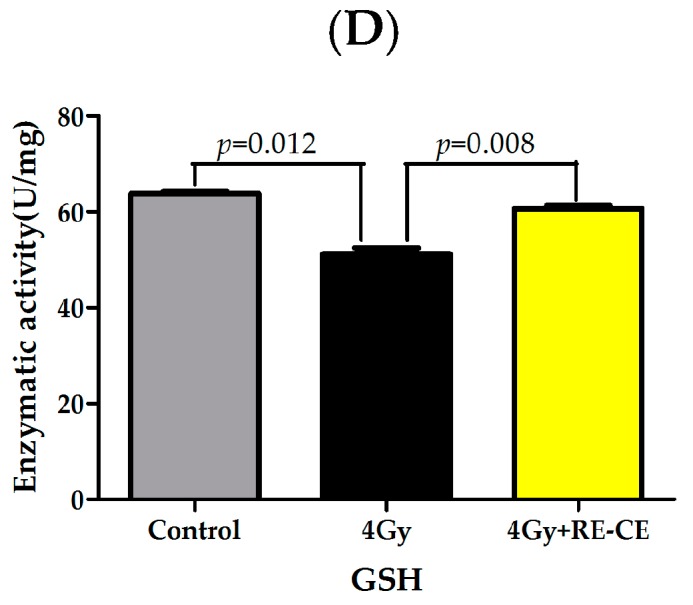
RE-CE ameliorates IR-induced repression of antioxidant enzymatic activities in c-kit positive cells. Mice were daily treated with the vehicle or RE-CE as described in the Materials and Methods. (**A**) Enzymatic activity of SOD in c-kit positive cells; (**B**) Enzymatic activity of GSH-PX in c-kit positive cells; (**C**) Enzymatic activity of CAT in c-kit positive cells. RE-CE treatment significantly increased the enzymatic activities of superoxide dismutase (SOD), glutathione peroxidase (GSH-PX), and catalase (CAT) in irradiated c-kit positive cells. The enzymatic activities are presented as means ± SEM (*n* = 3).

**Figure A6:**
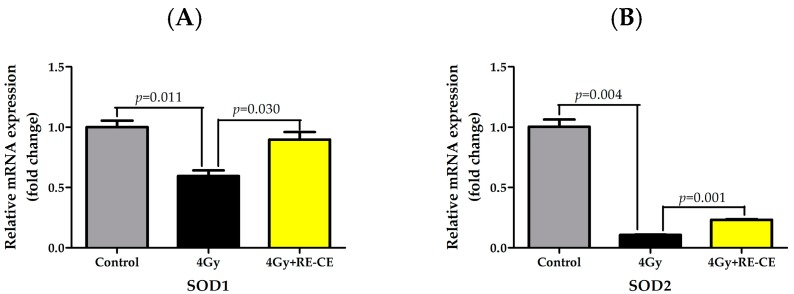

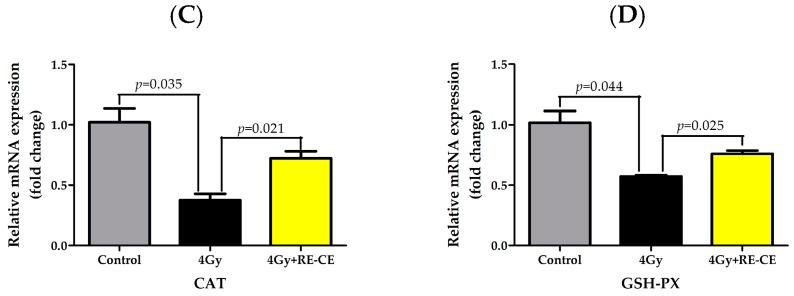
CE ameliorates IR-induced repression of antioxidant enzyme transcripts in c-kit positive cells. Mice were daily treated with the vehicle or CE as described in the Materials and Methods. (**A**) Relative mRNA expression of SOD1 in c-kit positive cells; (**B**) Relative mRNA expression of SOD2 in c-kit positive cells; (**C**)Relative mRNA expression of CAT in c-kit positive cells; (**D**) Relative mRNA expression of GSH-PX in c-kit positive cells. CE treatment significantly increased antioxidant enzyme transcripts in irradiated c-kit positive cells. The relative mRNA expression of SOD2, CAT, and GSH-PX are presented as means ± SEM (*n* = 3).

Antioxidant enzymes minimize the perturbations caused by ROS under normal conditions. However, the significantly elevated production of ROS under pathological conditions overcomes cellular levels of antioxidants, inducing oxidative stress. To assess the effects of RE-CE on antioxidant enzymes, we measured the enzymatic activity of SOD, GSH-PX, and CAT in c-kit positive cells. Moreover, we also measured the glutathione (GSH) level which is a global cellular antioxidant. The results showed that 4 Gy TBI down-regulated the enzymatic activities of SOD, GSH-PX, and CAT, and decreased the GSH level in c-kit positive cells, but RE-CE treatment dramatically ameliorated the repression of these enzymatic activities and increased the GSH level. (Figure 8). We also confirmed the increase in transcripts of SOD1, SOD2, CAT, and GSH-PX in irradiated c-kit positive cells after RE-CE treatment (Figure A6). These findings indicate that the effects of RE-CE treatment on IR-induced hematopoietic injury may be, at least in part, attributed to ROS scavenging and enhancing antioxidant enzymatic activities in irradiated c-kit positive cells.

### 2.9. RE-CE Improves Survival of Lethally Irradiated Mice

**Figure 9:**
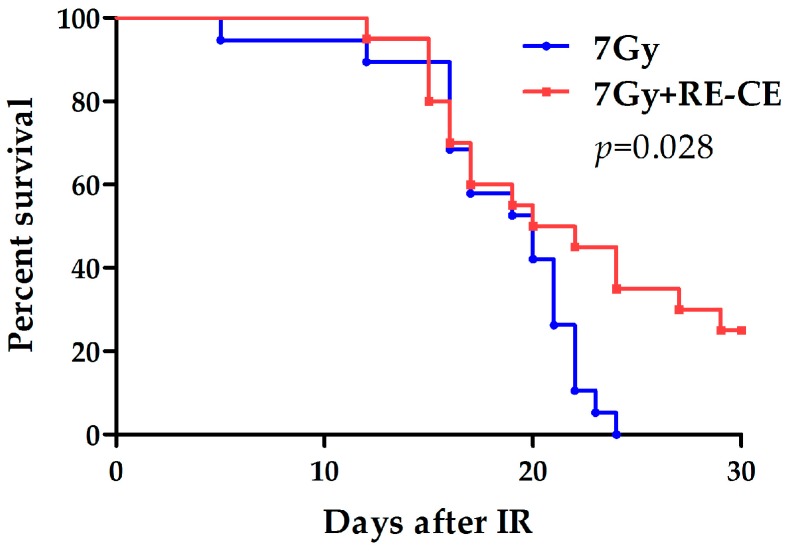
RE-CE improved survival of lethally irradiated mice. Mice were daily treated with the vehicle or 50 mg/kg RE-CE by gavage for 30 min before 7 Gy TBI and then 7 days after TBI. Curves show the survival rate of 30 days after exposure to a lethal dose of TBI. RE-CE treatment significantly increases the survival rate of 7 Gy irradiated mice (*n* = 20).

To test whether RE-CE affected the survival of irradiated mice, we fed mice with 50 mg/kg RE-CE or vehicle 30 min before radiation and up to 7 days after radiation. As shown in Figure 9, all vehicle-treated mice died within 24 days following 7 Gy TBI. However, 25% of RE-CE-treated mice were alive 30 days after TBI. These findings suggest that RE-CE treatment significantly increases the survival rate of irradiated mice.

### 2.10. Flavonoids Are Identified as the Major Component of Coriander Extract

**Figure 10:**
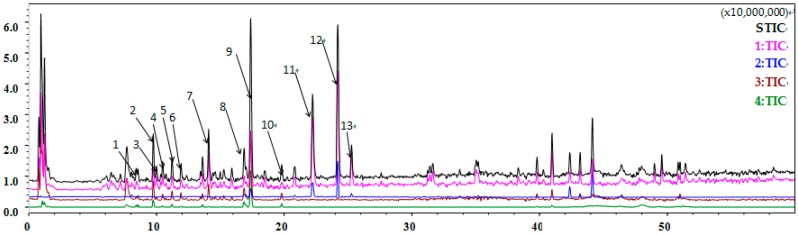
Total ion chromatography (TIC) analysis of coriander extract. liquid chromatography-mass spectrometry (LC/MS) analyses were performed using liquid chromatography to identify the active component of coriander extract. Reverse phase separation was performed at 40 °C using an ACOUITY UPLC BEH-C18 Column (1.7 μm). The mobile phase consisted of 0.1% formic acid in water (solvent A) and HPLC grade methanol (solvent B). The gradient program consisted of: 5% B for 0 min, 95% B for 50 min, and 95% B for 60 min. The eluted peaks were. 1: Chlorogenic acid; 2: Esculin or Daphnetin-8-*O*-glucoside; 3: Hydroxybenozyl Tryptophan; 4: Methoxycinnamic acid glucoside; 5: Ferulic acid glucoside; 6: Citroside A, Citroside B, or Icariside B2; 7: Coriandrone E hexoside; 8: Quercetin 3-glucuronide; 9: Rutin; 10: Nicotiflorin; 11: Dihydrocoriandrin; 12: Coriandrone A or B; 13: Coriandrin.

To identify the major component that functioned as a radioprotector for the hematopoietic system in coriander extract, we performed liquid chromatography-mass spectrometry (LC/MS) analyses using liquid chromatography. As shown in Figure 10 and Table 1, flavonoids, phenolic acids, and coumarins were the main components in coriander extract, with rutin, quercetin 3-glucuronide, and nicotiflorin as the major compounds in the flavonoids.

## 3. Discussion

Despite the wide use of coriander as a medicinal herb to treat various diseases, the therapeutic potential of coriander as a radioprotective agent is poorly understood. In this study, we explored whether RE-CE treatment could ameliorate IR-induced hematopoietic injury in a TBI mouse model. Our results indicated that RE-CE treatment improved the survival of irradiated mice, and rescued IR-induced injury in the spleen, thymus, and lung. It seems that there is no direct connection between IR-induced hematopoietic injury and lung injury; however, under our experimental conditions, radioprotective agents including RE-CE both protected the hematopoietic system from injury and alleviated IR-induced lung injury 14 days after 4 Gy TBI. A recent study reported that the lung is a site of platelet biogenesis and a reservoir for hematopoietic progenitors [28]. When hematopoietic stem cells decrease in the bone marrow, hematopoietic progenitors migrate out of the lung and reconstitute the bone marrow. In the light of these findings, one may speculate that RE-CE alleviates IR-induced HSPCs injury both in the bone marrow and lung, and HSPCs may play a role in IR-induced lung injury. Further work is required to establish the potential connection between IR-induced hematopoietic injury and lung injury.

Myelosuppression is one of the common symptoms of hematopoietic injury, mainly caused by the exhaustion and dysfunction of HSPCs. The RE-CE treatment mitigated IR-induced myelosuppression and promoted the recovery of HSPCs populations to provide adequate reserves for hematopoietic injury. It has been demonstrated that lymphoid-biased HSCs are more sensitive to IR-induced differentiation than myeloid-biased HSCs, resulting in an imbalance in myeloid-lymphoid differentiation in irradiated mice [26]. The RE-CE treatment mitigated myeloid skewing in irradiated mice and promoted balanced differentiation of irradiated HSPCs. IR may also impair the self-renewing ability of HSPCs, causing long-term or permanent damage to the hematopoietic system [29,30]. The RE-CE treatment not only mitigated IR-induced myelosuppression, but also promoted HSPCs colony forming abilities and engraftment. Together, we demonstrate that the RE-CE treatment ameliorates IR-induced HSPCs injury in cell number, differentiation function, and colony forming abilities.

To explore the underlying mechanisms, we measured cell apoptosis and DNA damage in LSKs and c-kit positive cells which are enriched with HSPCs. Results indicated that RE-CE treatment inhibited apoptosis and DNA damage in HSPCs, which may benefit the recovery of HSPCs populations and functions. Parsley, a similar herb to coriander in the *Apiaceae* family, has been reported to protect mouse fibroblasts from DNA damage induced by H_2_O_2_ and induce apoptosis of cancer cells [31,32]. We confirm the protective effects of RE-CE on DNA damage in irradiated HSPCs, and demonstrate for the first time that the RE-CE treatment inhibits IR-induced apoptosis of HSPCs. However, the mechanisms by which RE-CE protects HSPCs from apoptosis are unknown and further exploration is warranted.

Rutin, a bioactive flavonoid, was identified as a leading compound in coriander extract, consistent with previously reported findings [17]. Rutin has been reported to decrease oxidative stress by regulating oxidative stress related genes and proteins [33,34,35]. Quercetin, also identified as a component of RE-CE, together with rutin have antioxidant and radioprotective potential in mice exposed to γ-radiation [36,37,38]. These compounds may be largely responsible for the radioprotective effect of RE-CE on the hematopoietic system, observed in our studies. Our results indicate that RE-CE alleviates IR-induced HSPCs injury probably by reducing oxidative stress. As ROS may be the primary cause of DNA damage and apoptosis in HSPCs 14 days after radiation, they may also be partly responsible for the disturbance of proliferation and differentiation in irradiated HSPCs. Furthermore, Coriandrone A or B were also the major compounds in RE-CE, however, there are no reports about the biological and radioprotective effect of Coriandrone A or B, which is worthy of further exploration. Subsequent studies focusing on the effect of the individual or combined compounds identified in coriander extract are warranted.

## 4. Materials and Methods

### 4.1. Reagents

Anti-mouse CD34 FITC (clone RAM34), anti-mouse CD3 APC (clone145-2C11), anti-mouse CD117 (c-kit) APC (clone 2B8), and anti-mouse Ly-6 A/E (Sca1) CE/Cy7 (clone D7) were purchased from eBioscience (San Diego, CA, USA). Biotin anti-mouse CD4 (clone GK1.5), CE anti-mouse CD4 (clone GK1.5), CE anti-mouse/human CD45R/B220 (clone RA3-6B2), biotin anti-mouse/human CD45R/B220 (clone RA3-6B2), PerCP anti-mouse/human CD45R/B220 (clone RA3-6B2), PerCP anti-mouse/human CD11b (clone M1/70), FITC anti-mouse/human CD11b (clone M1/70), biotin anti-mouse/human CD11b (clone M1/70), PerCP anti-mouse Ly-6G/ Ly-6C(Gr1) (clone RB6-8C5), biotin anti-mouse Ly-6G/ Ly-6C(Gr1) (clone RB6-8C5), CE/Cy7 anti-mouse Ly-6G/Ly-6C(Gr1) (clone RB6-8C5), biotin anti-mouse Ter119 (clone TER119), FITC anti-mouse Ter119 (clone TER119), FITC anti-mouse CD8 (clone 53-6.7), biotin anti-mouse CD8 (clone 53-6.7), CE anti-mouse CD71 (clone RI7217), and PerCP streptavidin were obtained from Biolegend (San Diego, CA, USA). Anti-γH2AX rabbit monoclonal antibody was purchased from Cell Signaling Technology (Danvers, MA, USA). FITC goat anti-rabbit IgG was obtained from ZSGB-BIO Origene (Beijing, China).

### 4.2. Mice

Male C57BL/6 (CD45.2) mice were purchased from the Institute of Laboratory Animal Sciences, Chinese Academy of Medical Sciences and Peking Union Medical College (Beijing, China). Male C57BL/6 (CD45.1) mice were purchased from the Institute of Hematology and Blood Disease, Chinese Academy of Medical Sciences and Peking Union Medical College (Tianjin, China). Male C57BL/6 (CD45.1/45.2) mice were bred in the experimental animal center of the Institute of Radiation Medicine. Mice were used at approximately 6 to 8 weeks of age. Animal experiments in our study were approved by the Animal Care and Ethics Committee of the Institute of Radiation Medicine (Permit number 1505, 1 Jan 2015). The study was performed in accordance with the principles of the Institutional Animal Care and Ethics Committee guidelines.

### 4.3. TBI and RE-CE Administration

For evaluations of the organ index, histomorphology, peripheral blood cell counts, and HSPCs differentiation, mice were randomly divided into 4 groups, namely control, TBI, TBI + 25 mg/kg RE-CE, and TBI + 50 mg/kg RE-CE. For the other experiments, mice were also divided into 4 groups consisting of control, 50 mg/kg RE-CE, TBI, and TBI + 50 mg/kg RE-CE. Mice received 7 Gy TBI for the survival experiment, and 6 Gy or 4 Gy TBI in other experiments at a dosage rate of 0.99 Gy/min. Mice in control and RE-CE groups were sham-irradiated. CE was dissolved in distilled water (vehicle), and then administrated once a day by gavage 30 min before radiation and up to 7 days after radiation in RE-CE and TBI + RE-CE groups. Mice in the control and TBI groups received the same volume of vehicle for the same frequency and duration as those in CE or TBI + RE-CE groups. Mice were finally euthanized on the 14th or 60th day after TBI.

### 4.4. Weight, Organ Index, Counts of Splenocyte and Thymocyte, and HE Staining

The body weight of individual mice was measured at the 14th day after exposure to 4 Gy TBI. The spleen, thymus, and lung were removed and weighed. The organ index was calculated according to the following formula: Organ index = [organ weight (g)/body weight (g)] × 10. Single cell suspensions of the spleen and thymus obtained by mechanical trituration were filtered and cells were counted. Specimens from spleen, thymus, and lung tissue were fixed with 4% formalin, embedded with paraffin, serially sectioned, and stained with hematoxylin and eosin. Specimens from the femur were decalcified with microwave and 10% ethylenediaminetertraacetic acid (EDTA) before being embedded.

### 4.5. Peripheral Blood Cell Counts and Wright-Giemsa Staining

Blood was obtained via the orbital sinus and was collected in EDTA tubes. The cell counts of peripheral blood including WBC counts, and LY% and NE% were analyzed with a hematology analyzer (Nihon Kohden, Japan). A Wright-Giemsa staining kit (Solarbio life sciences, Beijing, China) was used according to the manufacturer’s instructions.

### 4.6. Isolation of BM Cells (BMCs) and Flow Cytometry Analysis

BM cells (BMCs) were isolated from tibias and femurs, suspended in phosphate-buffered saline (PBS), filtered, and counted prior to antibody staining. For B cell, T cell, and myeloid cell analysis in peripheral blood, 50 µL peripheral blood was harvested and incubated with premixed antibodies of B220, CD3, Gr1, and CD11b at room temperature for 30 min, and subsequently red blood cells were removed with BD FACSTM Lysing Solution. For B cell analysis in BM, a 1 × 10^6^ BMC suspension was incubated with CD3 and B220 antibodies at 4 °C for 30 min. For immature erythrocytes analysis in BM, a 1 × 10^6^ BMC suspension was incubated with CD71 and Ter119 antibodies at 4 °C for 30 min. For the analysis of HPSC frequency, 5 × 10^6^ BMCs were incubated with biotin-conjugated antibodies specific for Gr1, Ter119, CD11b, B220, CD8, and CD4 and then stained with streptavidin, sca1, c-kit, and CD34 antibodies. Data acquisition was performed on a BD Accuri C6 and analyzed by BD Accuri C6 software (BD Bioscience, San Jose, CA, USA).

### 4.7. CFU-GM and CFU-S Assays

For CFU-GM assay, 1 × 10^4^ BMCs from unirradiated mice and 1 × 10^5^ BMCs from irradiated mice were cultured in M3534 methylcellulose medium (Stem Cell Technologies, Vancouver, BC, Canada) for 5 days. The number of CFU-GM containing more than 30 cells was counted according to the manufacturer’s instructions, and the results are expressed as the number of CFU-GM per 10^5^ BMCs. For the CFU-S assay, the spleen was soaked in trinitrophenol for 6 h and then the number of CFU-S was counted.

### 4.8. Competitive Bone Marrow Transplantation

1 × 10^6^ BMCs from C57BL/6 (CD45.2) treated mice and 1 × 10^6^ BMCs from C57BL/6 (CD45.1/45.2) mice were mixed and transplanted into lethally irradiated C57BL/6 mice (CD45.1). The percentage of donor-derived (CD45.2) cells in the peripheral blood of recipients was examined 4 months after transplantation.

### 4.9. Isolation of c-Kit Positive Cells

BMCs were stained with c-kit APC antibody for 30 min on ice. Then the cells were washed with PBS, and incubated with anti-APC microbeads (Miltenyi Biotec, Teterow, Germany) for 15 min at room temperature. C-kit positive cells were sorted by MACS using a LS column in the QuadroMACS™ Separator (Miltenyi Biotec, Teterow, Germany).

### 4.10. Apoptosis Assay

1 × 10^6^ c-kit positive cells or 5 × 10^6^ BMCs stained with LSK (Gr1, Ter119, CD11b, B220, CD8, CD4, sca1 and c-kit) antibodies were prepared to perform the apoptosis assay with an Annexin V-FITC Apoptosis Detection Kit (BD Biosciences, San Jose, CA, USA) according to the manufacturer’s protocol. Samples were collected by a BD Accuri C6 and analyzed using the BD Accuri C6 software (BD Bioscience, San Jose, CA, USA).

### 4.11. Analysis of γH2AX Staining in c-Kit Positive Cells

1 × 10^6^ c-kit positive cells or 5 × 10^6^ BMCs stained with LSK antibodies were fixed and permeabilized with BD Cytofix/Cytoperm buffer for 30 min at room temperature. After washed with BD perm/Wash buffer twice, cells were incubated with anti-γH2AX (1:100) for 1 h and then stained with FITC goat anti-rabbit IgG for 30 min at room temperature. The MFI of γH2AX in c-kit positive cells was detected by flow cytometry.

### 4.12. Analysis of Intracellular ROS Levels

1 × 10^6^ c-kit positive cells or 5 × 10^6^ BMCs stained with LSK antibodies were stained with 2,7-dichlorodihydrofluorescein diacetate (DCFDA, Beyotime Biotechnology, Nanjing, China; 10 μΜ), MitoSox (Life Technologies, Grand Island, NY, USA; 10 μM), and dihydroethidium (DHE, Beyotime Biotechnology, Nanjing, China; 5 μM) for 20 min, 30 min, and 10 min, respectively, in a 37 °C water bath. The intracellular ROS levels of c-kit positive cells were analyzed by measuring the MFI of DCF, Mitosox, and DHE using a flow cytometer.

### 4.13. Analysis of SOD, GSH-PX, GSH, and CAT Activities

SOD, GSH-PX, GSH, and CAT enzymatic activities of c-kit positive cells were determined using the total superoxide dismutase assay kit with WST-8, total glutathione peroxidase assay kit, total glutathione assay kit, and catalase assay kit (Beyotime Institute of Biotechnology, Nanjing, China), respectively, according to the manufacturer’s instructions. Briefly, 1 × 10^6^ c-kit positive cell lysate or homogenate was added into the detection buffer, and the maximum absorption wavelength was examined at 450, 340, 412, and 520 nm by the colorimetric method for the detection of enzymatic activities of SOD, GSH-PX, GSH, and CAT, respectively.

### 4.14. Quantitative Real-Time PCR

The total RNA of c-kit positive cells was extracted with TRIzol reagent (Life Technologies, Grand Island, NY, USA). Reverse transcription was performed with a Revert Aid First Strand cDNA Synthesis Kit (Thermo Scientific, Waltham, MA, USA), according to the manufacturer’s instructions. All PCRs were conducted under an ABI 7500 Sequence Detection System and GAPDH (Thermo, Waltham, MA, USA) was used as control. Three pairs of primers were designed for SOD2, CAT, and GSH, according to the sequences shown in a previous study [39]. The forward primer sequence of SOD1 was AACCAGTTGTGTTGTCAGGAC, and its reverse primer sequence was CCACCATGTTTCTTAGAGTGAGG.

### 4.15. Preparation and Component Identification of CE

RE-CE was purchased from ICTORY Biological Technology Co., Ltd. (Xi’an, China). The raw materials of coriander herbs were dried without access to direct sunlight after copious washing in running water. The dried materials were ground into a fine power and passed through a 40-mesh sieve. This powered product (500 g) was submitted to extraction with aquaous ethanol (30%, 5 L) in a shaker at 60 °C for 2 h. The extract waste was filtered out and the solution was concentrated by a rotary evaporator and dried under vacuum. The extract was stored at 4 °C for further use. LC/MS analyses were performed on a Shimadzu LC-30AD liquid chromatography interfaced with an IT-TOF mass spectrometer (Shimadzu Corp., Kyoto, Japan) with electrospray ionization. An ACOUITY UPLC BEH-C18 Column (1.7 μm) was used. The column temperature was 40 °C and the elution velocity was 0.3 mL/min. The sample extracts were analyzed using a gradient program, and the mobile phase consisted of 0.1% formic acid in water (solvent A) and HPLC grade methanol (solvent B). The gradient program consisted of: 5% B for 0 min, 95% B for 50 min, and 95% B for 60 min. The nebulizer gas flow rate was 1.5 L/min. CDL and block heater temperatures were both 200 °C. The spray and detector voltages were 4.5 and 1.58 kV, respectively. The scan time and mass range were 1.5 s and 50–1000 *m*/*z*, respectively. The injected volume was 2 μL.

### 4.16. Statistical Analysis

Statistical analysis was performed using GraphPad Prism 5 software (GraphPad Prism Software Inc., La Zolla, CA, USA) with an unpaired *t* test (two-tails) and Welch’s correction *t*-test for mean comparisons. Survival rates were analyzed with the Kaplan Meier method and Log rank test. Data are presented as means ± SEM, and differences were considered statistically significant at *p* < 0.05.

## 5. Conclusions

Our study demonstrates for the first time that RE-CE treatment protects mice from IR-induced hematopoietic injury. RE-CE treatment improves the proliferation and differentiation function, and inhibits apoptosis and DNA damage in irradiated HSPCs. Our studies also suggest the potential of RE-CE to scavenge ROS and enhance antioxidant enzyme activities in irradiated HSPCs. Further studies are warranted to explore the effects of RE-CE as a radioprotective agent to ameliorate IR-induced injuries.

## Figures and Tables

**Table 1:** Identified constituents of the coriander extract obtained from the aerial parts of *Coriandum sativum* L.

Peak Number	Retention Time (min)	Molecular Ion Peak *m*/*z*	Ion Mode	Molecular Formula	Compound Type	Compound Name
1	8.452	377.0836	[M + Na]+	C_16_H_18_O_9_	Phenolic acid	Chlorogenic acid
		353.0790	[M − H]−			
2	9.735	363.0687	[M + Na]+	C_15_H_16_O_9_	Coumarin	Esculin or Daphnetin-8-*O*-glucoside
		339.0614	[M − H]−			
3	10.010	371.1245	[M + HCOO]−	C_18_H_18_N_2_O_4_	Animo acid	Hydroxybenozyl tryptophan
4	10.487	365.1189	[M + Na]+	C_18_H_22_O_8_	Phenolic acid	Methoxycinnamic acid glucoside
		341.1163	[M − H]−			
5	11.220	379.0975	[M + Na]+	C_16_H_20_O_9_	Phenolic acid	Ferulic acid glucoside
		355.0943	[M − H]−			
6	11.917	409.1818	[M + Na]+	C_19_H_30_O_8_	Norcarotenoid	Citroside A or B or Icariside B2
		431.1830	[M + HCOO]−			
7	14.080	433.1079	[M + Na]+	C_19_H_22_O_10_	Coumarin	Coriandrone E hexoside
		455.1075	[M + HCOO]−			
8	16.848	479.0805	[M + H]+	C_21_H_18_O_13_	Flavonoid	Quercetin 3-glucuronide
		477.0564	[M − H]−			
9	17.398	611.1590	[M + H]+	C_27_H_30_O_16_	Flavonoid	Rutin
		609.1390	[M − H]−			
10	19.800	617.1465	[M + Na]+	C_27_H_30_O_15_	Flavonoid	Nicotiflorin
		593.1367	[M − H]−			
11	22.220	255.0614	[M + Na]+	C_13_H_12_O_4_	Coumarin	Dihydrocoriandrin
12	24.200	293.1311	[M + H]+	C_16_H_20_O_5_	Coumarin	Coriandrone A or B
13	25.282	253.0453	[M + Na]+	C_13_H_10_O_4_	Coumarin	Coriandrin

## Abbreviations

IR	ionizing radiation
HSPCs	hematopoietic stem and progenitor cells
CE	coriander extract
ROS	reactive oxygen species
BM	bone marrow
TBI	total body irradiation
SOD	superoxide dismutase
GSH-PX	glutathione peroxidase
CAT	catalase
WBC	white blood cell
LY	lymphocyte
NE	neutrophil
BMCs	bone marrow cells
HPCs	hematopoietic progenitor cells
CFU-S	spleen colony-forming units
CFU-GM	colony of granulocyte macrophage cells
